# Complement System in the Pathogenesis of Benign Lymphoepithelial Lesions of the Lacrimal Gland

**DOI:** 10.1371/journal.pone.0148290

**Published:** 2016-02-05

**Authors:** Jing Li, Xin Ge, Xiaona Wang, Xiao Liu, Jianmin Ma

**Affiliations:** 1 Beijing Tongren Eye Center, Beijing Tongren Hospital, Capital Medical University, Beijing Ophthalmology and Visual Sciences Key Laboratory, Beijing, China; 2 Beijing Institute of Ophthalmology, Beijing Tongren Hospital, Capital Medical University, Beijing, China; Sichuan University, CHINA

## Abstract

**Objective:**

We aimed to examine the potential involvement of local complement system gene expression in the pathogenesis of benign lymphoepithelial lesions (BLEL) of the lacrimal gland.

**Methods:**

We collected data from 9 consecutive pathologically confirmed patients with BLEL of the lacrimal gland and 9 cases with orbital cavernous hemangioma as a control group, and adopted whole genome microarray to screen complement system-related differential genes, followed by RT-PCR verification and in-depth enrichment analysis (Gene Ontology analysis) of the gene sets.

**Results:**

The expression of 14 complement system-related genes in the pathologic tissue, including *C2*, *C3*, *ITGB2*, *CR2*, *C1QB*, *CR1*, *ITGAX*, *CFP*, *C1QA*, *C4B|C4A*, *FANCA*, *C1QC*, *C3AR1 and CFHR4*, were significantly upregulated while 7 other complement system-related genes, *C5*, *CFI*, *CFHR1|CFH*, *CFH*, *CD55*, CR1L and *CFD* were significantly downregulated in the lacrimal glands of BLEL patients. The microarray results were consistent with RT-PCR analysis results. Immunohistochemistry analysis of C3c and C1q complement component proteins in the resected tissue were positive in BLEL patients, while the control group had negative expression of these proteins. Gene ontology (GO) analysis revealed that activation of the genes of complement system-mediated signaling pathways were the most enriched differential gene group in BLEL patients.

**Conclusions:**

Local expression of complement components is prominently abnormal in BLEL, and may well play a role in its pathogenesis.

## Introduction

Benign lymphoepithelial lesion (BLEL), also known as Mikulicz disease, was first reported by the Polish scholar Mikuliz in 1888[1]. BLEL is uncommonly seen, with the major clinical manifestations being symmetrical and painless enlargement of the bilateral lacrimal glands and/or the salivary glands[2–3]. Currently, the cause and pathogenesis of BLEL remain unclear. Studies have shown that BLEL belongs to the category of IgG4-related diseases[4–5]. Clinically, BLEL can be treated with glucocorticoid therapy, but the effects are not ideal. Research into the etiology and pathology of BLEL should help enhance the development of treatments that target the disease.

The complement system is part of the innate immune system that is important in clearing infectious pathogens[6]. It has been demonstrated that complement system abnormalities play roles in certain IgG4-related disease, as well as in a variety of eye diseases, including keratitis, uveitis, and diabetic retinopathy[7–10]. Whether a link exists between the complement system and the BLEL remains unknown. In this study, we employed microarray technology to examine the local tissue expression of the complement system in the setting of BLEL of the lacrimal gland.

## Materials and Methods

### Subjects

A total of 9 patients with lacrimal gland BLEL were registered and treated at the Beijing Tongren Hospital affiliated to Capital Medical University between August 2010 and March 2013. The study population comprised two males and seven females, the ages ranged from 36 to 70 years with a median age of 46 years. All BLEL diagnosis was confirmed by post-surgical histological examinations. Nine subjects with orbital cavernous hemangioma were recruited as a control group comprised of 3 male and 6 female subjects, aged between 31 and 59 years. Informed written consent was obtained from all subjects. The research was approved by the Ethics Committee of Beijing Tongren Hospital, Capital Medical University and conducted according to the principles in the declaration of Helsinki. Written informed consent was obtained from all participants.

### Tissues

Specimens from BLEL patients and orbital cavernous hemangioma patients were collected during surgery. Each specimen was divided into two; one half was stored in liquid nitrogen until genetic analysis was performed, and the second half was fixed in 10% formalin and paraffin-embedded within 24 hours of harvesting.

### Instruments and Reagents

Human genome microarray (version 5.0; Phalanx Biotech Group); RNA amplification kit (New England Biolabs, Ipswich, MA, USA); Agilent gene hybridization buffer kit (New England Biolabs, Ipswich, MA, USA); hybridization kit (New England Biolabs, Ipswich, MA, USA); RNA extraction and reverse transcription kit (New England Biolabs, Ipswich, MA, USA) were used in this study.

### Microarrays

RNA was extracted from frozen tissues using Trizol reagent (New England Biolabs, Ipswich, MA, USA). Total RNA was detected by agarose gel electrophoresis and lab-on-a-chip electrophoresis. Total RNA was purified using Qiagen Mini Kit. The absorbance ratio, A260/280,of 1.7–2.1 indicates that the quality of RNA is adequate to be applied in the array experiments. The 1st and 2nd strand of cDNA were synthesized by a one-step reaction. The cRNA samples were fluorescently cy5 labeled and concentration was determined using Qiagen Mini Kit through calculation of incorporation of fluorescent molecules. Then cRNA samples were fragmented. Duplicate chips were used for each RNA sample. The chips were then hybridized and washed. Each array was scanned using the GenePix 1 4000B microarray scanner (Axon Instruments Inc., Union City, California, USA) and analysis was carried out using GeneSpring software (Agilent, Santa Clara, California, USA).

### Reverse Transcription (RT)-PCR

We randomly selected 3 differentially expressed genes as verification genes and designed the corresponding PCR primers. Primer information is shown in **Table 1**. Following the manufacturer’s instructions of the RT kit, we synthesized cDNA from the extracted total RNA using reverse transcription, and used it as a template to perform RT-PCR. The PCR reaction conditions were as follows: 95°C 10min; 95°C 30s; 58°C/60°C/60°C/60°C/56°C 30s: 72°C 30s; 30 cycles, and *GAPDH* was used as the internal reference.

**Table 1:** Differentially expressed genes of the complement System in BLEL.

Gene Symbol	Description	Changes	P Value
**C3**	complement component 3	up	2.18E-21
**C2**	complement component 2	up	9.40E-13
**ITGB2**	integrin, beta 2 (complement component 3 receptor 3 and 4 subunit)	up	5.21E-11
**CR2**	complement component (3d/Epstein Barr virus) receptor 2	up	1.76E-06
**C1QB**	complement component 1, q subcomponent, B chain	up	1.18E-10
**CR1**	complement component (3b/4b) receptor 1 (Knops blood group)	up	1.69E-10
**ITGAX**	integrin, alpha X (complement component 3 receptor 4 subunit)	up	4.38E-09
**CFP**	complement factor properdin	up	5.83E-06
**C1QA**	complement component 1, q subcomponent, A chain	up	8.90E-06
**C4B|C4A**	complement component 4B (Chido blood group)|complement component 4A (Rodgers blood group)|complement C4-B-like	up	1.20E-05
**FANCA**	Fanconi anemia, complementation group A	up	6.45E-04
**C1QC**	complement component 1, q subcomponent, C chain	up	0.0015
**C3AR1**	complement component 3a receptor 1	up	0.0085
**CFHR4**	complement factor H-related 4	up	9.40E-13
**C5**	complement component 5	down	3.82E-09
**CFI**	complement factor I	down	2.03E-12
**CFHR1|CFH**	complement factor H-related 1|complement factor H	down	3.61E-13
**CFH**	complement factor H	down	1.61E-13
**CD55**	CD55 molecule, decay accelerating factor for complement (Cromer blood group)	down	8.46E-08
**CR1L**	complement component (3b/4b) receptor 1-like	down	1.95E-07
**CFD**	complement factor D (adipsin)	down	0.0059

### Immunohistochemical staining

Orbital lesion tissue slices were dewaxed, incubated in 3% H_2_O_2_ at room temperature for 5 to 10 minutes, washed with distilled water, and soaked in PBS for 5 minutes. Tissues were blocked with 10% normal goat serum at room temperature for 10 minutes before primary antibodies (C3c, Abcam, ab204121; C1q, Abcam, ab71089) were added and incubated overnight at 4°C. Tissues were then washed three times for 5 minutes with PBS before the drop-wise addition of biotin-labeled secondary antibody and incubated at 37°C for 30 minutes. Tissues were again washed three times for 5 minutes with PBS and the slices were stained using DAB reagent, washed with water, counter-stained with hematoxylin, mounted, and imaged under a microscope (OLYMPUS CX41, JAPAN).

### Data processing and statistical analysis

Using Bayesian Limma algorithms, we conducted analysis of differential gene expression in the experimental BLEL group and the control group. We also conducted functional significance analysis of differential genes using Fisher method. By means of the Gene Ontology (GO) database, we analyzed classification of differential gene functions, and carried out gene enrichment analysis. *P* < 0.05 was set as the criteria for statistical significance. Using SPSS 17.0 software, paired *t* test was adopted to compare differences in gene expression levels, and *P* < 0.05 was set as the criteria of statistical significance.

## Results

### Complement System-related Differential Gene Expression in BLEL

Test results from both groups were compared using DifferGene program, *P* < 0.05 and FC>2 were set as selection criteria. Expression of 14 complement system-related differential genes, including *C2*, *C3*, *ITGB2*, *CR2*, *C1QB*, *CR1*, *ITGAX*, *CFP*, *C1QA*, *C4B|C4A*, *FANCA*, *C1QC*, *C3AR1 and CFHR4* were significantly upregulated while that of 7 complement system-related differential genes, which were *C5*, *CFI*, *CFHR1|CFH*, *CFH*, *CD55*, CR1L and *CFD* were remarkably downregulated (Table 1).

### Verification of Differential Expression by RT-PCR

**Fig 1:**
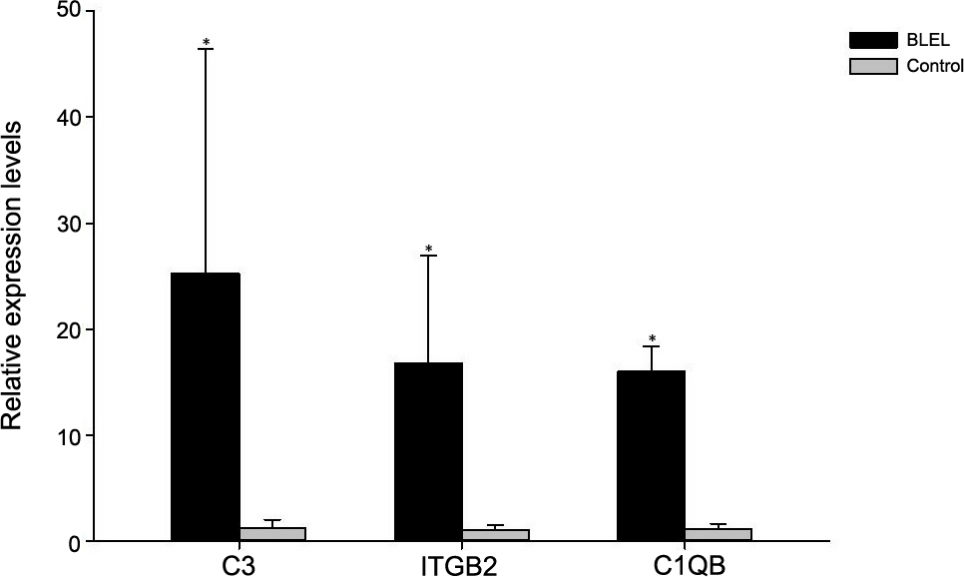
3 genes that were upregulated as identified by microarray data, were subjected to RT-PCR analysis for confirmation purposes. The results of differences in gene expression from microarray and RT-PCR were found to be consistent.

We randomly selected 3 genes with differential expression evidenced in the microarrays for experimental confirmation using RT-PCR analysis. The results of differences in gene expression from microarray and RT-PCR were found to be consistent (Table 2 and Fig 1).

**Table 2:** Sequences of real-time PCR primers.

Gene	Genebank accession	Primer Sequence 5’→3'	Amplicon (bp)
**GAPDH**	NM_002046.4	F: TGTTGCCATCAATGACCCCTT	202
		R: CTCCACGACGTACTCAGCG	
**C3**	NM_000064.2	F: CCTGGACAAGGTCTCACACTC	136
		R: GAACCGGGTACAGCTTTCCT	
**ITGB2**	NM_001127491.1	F: ACCCCAAGTTTGCTGAGAGT	111
		R: ATCCTCAAGAGCTGTGGCAA	
**CIQB**	NM_000491.3	F: ATGCCTACAACACCTTCCAGG	150
		R: CTGGAAAGAGCAGGAACCCG	

### Expression of C3c and C1q Complement Components in BLEL tissues

**Fig 2:**
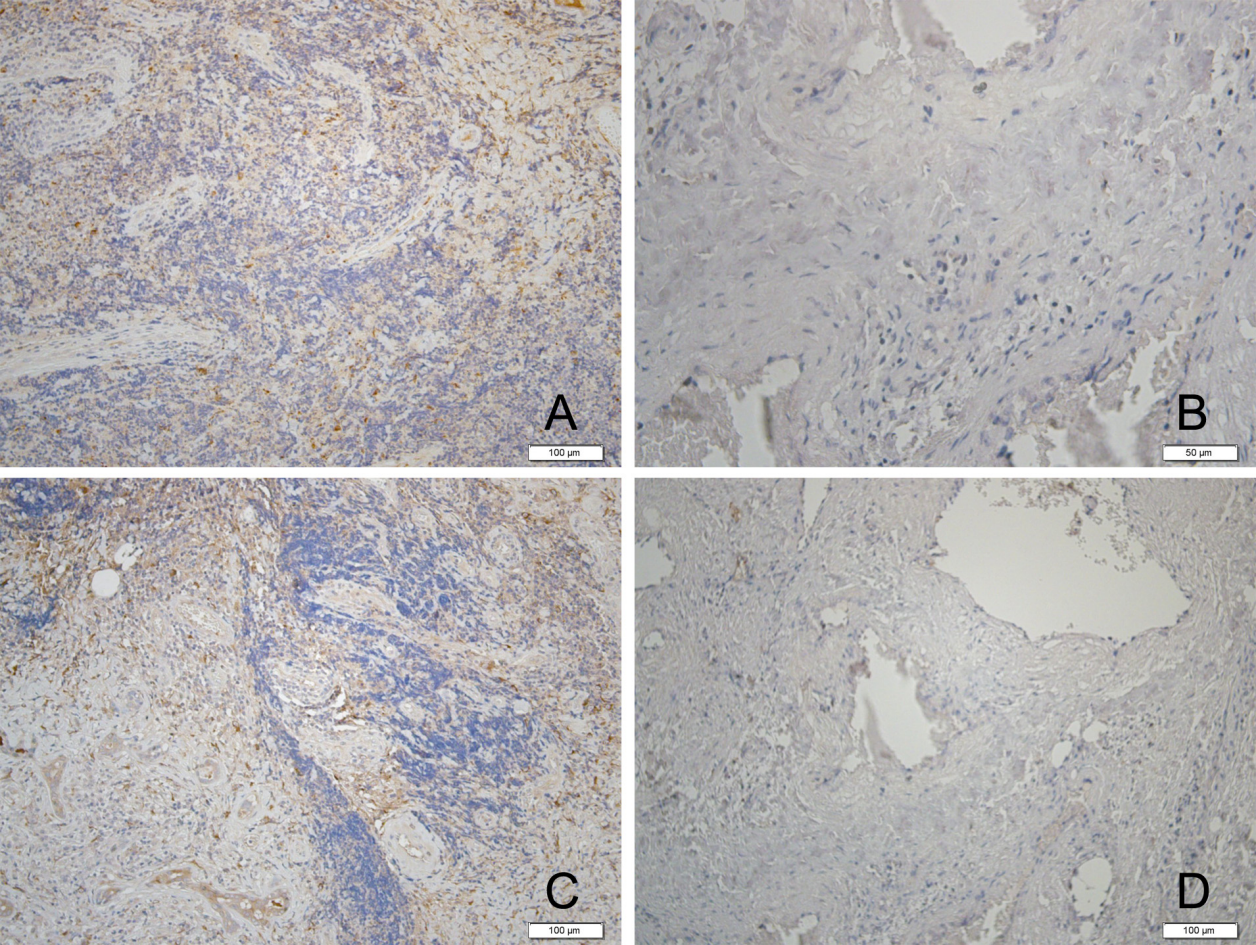
Both C3c (A) and C1q (C) expressions were positive in the BLEL samples (black arrows), while negative in the control group (B and D).

We performed immunohistochemical staining to assess the expression of C3c and C1q complement components in tissues. Both C3c and C1q expressions were positive in the BLEL samples, while negative in the control group (Fig 2).

### GO Enrichment Analysis of Complement System-related Genes in BLEL

**Fig 3:**
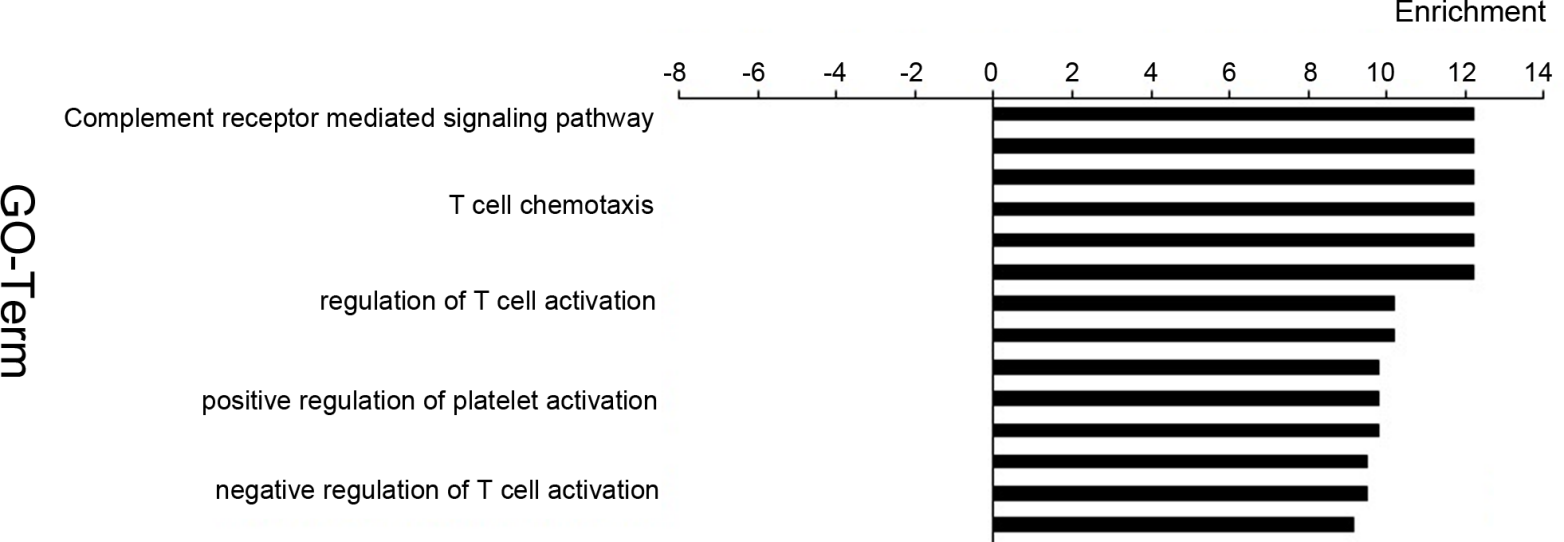
GO analysis showed that the most enriched and activated functional gene group in the differential genes of BLEL includes genes related to signaling pathways mediated by the complement system.

In our GO analysis, we found the most enriched and activated functional gene group in the differential genes of BLEL includes genes related to signaling pathways mediated by the complement system (Fig 3).

## Discussion

In recent years, IgG4-related diseases have become recognized as a new clinical disease entity. One such IgG4-related disease is autoimmune pancreatitis, which can also affect the parotid gland, bile duct, liver, lungs and lymph nodes[1]. Increased levels of IgG4 detected by serological tests and/or increased IgG4-positive plasma cells indicated by immunohistochemical staining are features of IgG4-related diseases[1]. At present, there is increasing evidence that BLEL is an IgG4-related disease[2–5].

A few recent studies have discovered the presence of abnormalities of the complement system in IgG4-related diseases, although the case numbers are small and not thoroughly persuasive[11–13]. A research group in Japan reported four IgG4-related diseases: autoimmune pancreatitis (AIP), retroperitoneal fibrosis, interstitial lung disease, and benign lymphoepithelial lesion. All of these IgG4-related diseases were accompanied with hypocomplementemia; to wit, reduced serum levels of complement C3 with elevated levels of complement C3 within lesions. Subsequently, they studied the serum CH50 in patients and found that increased serum CH50 was associated with improvement of disease condition while decreased serum CH50 indicated aggravation of disease conditions. Thus, it has been argued that serum levels of complement system components may be biological markers of IgG4-related diseases[11]. Muraki et al. reported that in 44 AIP patients, low C3 and C4 were observed in 36%, and low CH50 was observed in 17% of patients[12]. Hypocomplementemia was observed in 22 of 41 patients with IgG4-related kidney disease, 16 of whom had low C3, C4, and CH50 levels in sera[13]. Furthermore, immune complex (IC) formation appeared to have a role in pathophysiology of the disease[14–15].

In this study, we applied microarray technology to study the local expression of the complement system genes in the setting of BLEL. Our results show that the expression of 14 complement system-related genes, including *C2*, *C3*, *ITGB2*, *CR2*, *C1QB*, *CR1*, *ITGAX*, *CFP*, *C1QA*, *C4B|C4A*, *FANCA*, *C1QC*, *C3AR1 and CFHR4*, were significantly upregulated in the resected BLEL tissue while that of 7 complement system related differential genes, including *CFH|CFHR1*, *CFH*, *CFI*, *CFHR1|CFH*, *C5*, *CD55*, and *CD59* were remarkably downregulated in BLEL tissues. Through GO analysis, microarray results showed that the most enriched and activated functional gene group in the differential genes of BLEL includes genes related to signaling pathways mediated by the complement system. Meanwhile, immunohistochemical analyses of C3c and C1q complement components provided confirmation at the protein level of the involvement of complement components in the affected tissue, and indeed the control group was negative for these proteins. Our results suggest a connection may exist between abnormalities of the local production of complement components and IgG4-related diseases. In other words, we hypothesize that the local activation of complement system may be a key factor in the pathogenesis of BLEL. Through direct or indirect effects, the complement system may trigger immune responses disorders, resulting in immunoreactive lesions in BLEL.

A limitation of this study is that it was difficult to collect truly normal lacrimal gland tissues due to ethical reasons, and therefore we chose orbital cavernous hemangioma as the control group. Conceivably, by proximity to the hemangioma, tissue level changes in complement could result in this otherwise normal lacrimal tissue, and we would not be able to assess this in our study design. Additionally, there are many diseases that are associated with lymphoepithelial lesions, including automimmune and lymphoproliferative diseases that may contribute to systemic production of complement. It is not certain that these potential underlying disorders are entirely ruled out in our patient population. Systemic production of complement may have contributed to tissue levels assessed with our immunohistochemistry.

In summary, this is the first study using whole-genome microarray to explore the effects of local expression of complement system genese in the pathogenesis of BLEL. Our study provides a new direction for study of disease pathogenesis and suggests new clues for the pathogenesis of IgG4-related diseases, including BLEL.

## Supporting Information

S1 PLOS ONE Clinical Studies Checklist (DOTX)
